# 24.4 COGNITION AND COMMUNICATION AS DETERMINANTS OF ADAPTIVE DEFICITS IN LATE LIFE SCHIZOPHRENIA

**DOI:** 10.1093/schbul/sby014.099

**Published:** 2018-04-01

**Authors:** Philip Harvey, Anjana Muralidharan

**Affiliations:** 1 Leonard M. Miller School of Medicine, University of Miami; 2 Baltimore VA Medical Center

## Abstract

**Background:**

Older adults with schizophrenia experience poor community integration and social functioning. These individuals are at elevated risk for functional decline and early institutionalization in long-term care facilities. Deficits in thought, language, and communication are core features of schizophrenia and may worsen with age; however, little research focuses on the functional sequelae of these impairments among older adults with schizophrenia.

**Methods:**

The present study examined the relationships among age, TLC deficits, and functional outcomes in a sample of community-dwelling middle-aged and older adults with schizophrenia (N=245; ages 40–85). Participants completed assessments of symptoms, neurocognition, TLC deficits, and functional outcomes. Two different categories of TLC deficits were examined: verbal underproductivity (i.e., alogia) and disconnected speech.

**Results:**

Regression analyses found that disconnected speech predicted impaired occupational functioning, while verbal under productivity predicted capacity to communicate skillfully in semi-structured social situations, as well as community functioning across interpersonal, occupational, and everyday living domains. Exploratory mediation analyses found that cognitive impairments were mediated by disconnected speed but not under productivity on certain functional outcomes.

**Discussion:**

Targeted training to improve TLC deficits, especially verbal underproductivity, among older adults with schizophrenia could have downstream effects on community functioning, improving outcomes for a vulnerable group. It is likely that cognitive training interventions would also facilitate these interventions.

